# Development of Liposome-Based Immunoassay for the Detection of Cardiac Troponin I

**DOI:** 10.3390/molecules26226988

**Published:** 2021-11-19

**Authors:** Remya Radha, Mohammad Hussein Al-Sayah

**Affiliations:** Department of Biology, Chemistry, and Environmental Sciences, American University of Sharjah, Sharjah 26666, United Arab Emirates; rradha@aus.edu

**Keywords:** myocardial infarction, cardiac troponin, cTnI-detection, calcein liposomes, fluorescence, signal quantification

## Abstract

Cardiovascular diseases (CVDs) are one of the foremost causes of mortality in intensive care units worldwide. The development of a rapid method to quantify cardiac troponin I (cTnI)—the gold-standard biomarker of myocardial infarction (MI) (or “heart attack”)—becomes crucial in the early diagnosis and treatment of myocardial infarction (MI). This study investigates the development of an efficient fluorescent “sandwich” immunoassay using liposome-based fluorescent signal amplification and thereby enables the sensing and quantification of serum-cTnI at a concentration relevant to clinical settings. The calcein-loaded liposomes were utilized as fluorescent nano vehicles, and these have exhibited appropriate stability and efficient fluorescent properties. The standardized assay was sensitive and selective towards cTnI in both physiological buffer solutions and spiked human serum samples. The novel assay presented noble analytical results with sound dynamic linearity over a wide concentration range of 0 to 320 ng/mL and a detection limit of 6.5 ng/mL for cTnI in the spiked human serum.

## 1. Introduction

Among the non-communicable diseases, cardiovascular diseases (CVDs) are among the leading causes of death in developing countries, including Mediterranean and Gulf Cooperation Council (GCC) regions [1,2,3]. The World Health Organization (WHO) cautioned on the high CVDs-related mortality of more than 23.6 million people by the year 2030 [4]. The fatty deposits and blockage of arteries lead to deadly acute myocardial infarction (MI), or heart attack, and the diagnosis for MI fails if the patients are asymptomatic, without chest pain or shortness of breath. Since there is no single diagnostic tool available for MI detection, diagnosis follows a regular clinical collection of the patient’s medical history, physical check-ups, and clinical testing [1,5]. Electrocardiography (ECG) is a common tool for the initial evaluation and spotting of MI patients; however, more than 40% of the cases show a normal ECG profile while testing [6,7]. Hence, worldwide research attention has moved to the measurement of certain biomarkers in the patient’s blood that could provide an accurate diagnosis of MI [8].

Troponin is a complex of three proteins (Troponin I (TnI), Troponin C (TnC), and Troponin T (TnT)) that regulate the contraction of the skeletal and cardiac muscles. The cardiac forms of TnI and TnT are released from myocardial cells during necrosis or cell death [9]. As cTnI is confined inside the heart muscle, its specificity towards MI detection stands significantly higher compared to other heart biomarkers [10]. In the event of MI, cTnI and cTnT are released from the injured heart muscles into the bloodstream and their concentration remains high over several days, even when no other symptoms of MI are present [11,12]. The normal concentration of cTnI is 1–2 ng/mL or less; after the onset of MI, the concentration of cTnI increases to about 50 ng/mL within 3–6 h, reaching as high as 500 ng/mL [13,14]. The precise quantification of the blood–cTn levels in a patient with ischemia/chest pain indicates whether the patient had a myocardial infarction (MI) or not [15,16]. In fact, troponin time curves indicate that the peak elevation of cTnI in the patient’s blood reflects the time and the magnitude of the MI [17,18]. For a small MI, the elevation peaks within 18 to 24 h and goes back to near normal within a couple of days, while, for a medium MI, a higher peak of cTnI is expected within 24 to 36 h and it will take more than five days to go to near normal levels. A much higher peak is expected for a strong MI within 36 to 48 h and it will take more than a week to reach near normal levels [17,18]. However, it should be noted that the temporary elevation of cTnI was also reported in cases of non-cardiac conditions, such as exercise-induced elevation [19,20], non-coronary diseases [21], chronic kidney diseases [22], critical illness pulmonary embolism, sepsis, and stroke [23]. Therefore, while the cTnI level is an important marker for the diagnosis of MI, considering other physical symptoms and the medical history of the patient are essential elements of good clinical practice.

Over the last couple of decades, the use of immunoassays for clinical and research purposes has gained remarkable attention due to their ability to accurately detect specific antigen or antibody molecules within a short span of time. There are different strategies used for the development of immunoassays; however, the “sandwich” immunoassay is one of the most reported assay designs for the detection of cTnI in the last few years [13,15,24,25,26]. This assay design is based on capturing the targeted antigen between a “capture” antibody and a “detecting” antibody where each binds at a different and a distant epitope. The capturing antibody is usually immobilized on a surface, while the detecting antibody is associated with the signaling mechanism and signal amplification of the detection process. Different signaling mechanisms have been reported, and these include optical (colorimetric, fluorescence, emission), electrochemical, and magnetic signals, in addition to radioactivity and surface plasmon resonance (SPR) [13,27,28,29,30,31,32,33,34,35,36,37,38,39,40,41,42]. Yet, many of these assay types require lab settings with large equipment and lengthy procedures, limiting their use at point of care. In addition, many of these assays suffer from either low sensitivity or a narrow range of detection, which limits their application in clinical settings.

**Scheme 1:**
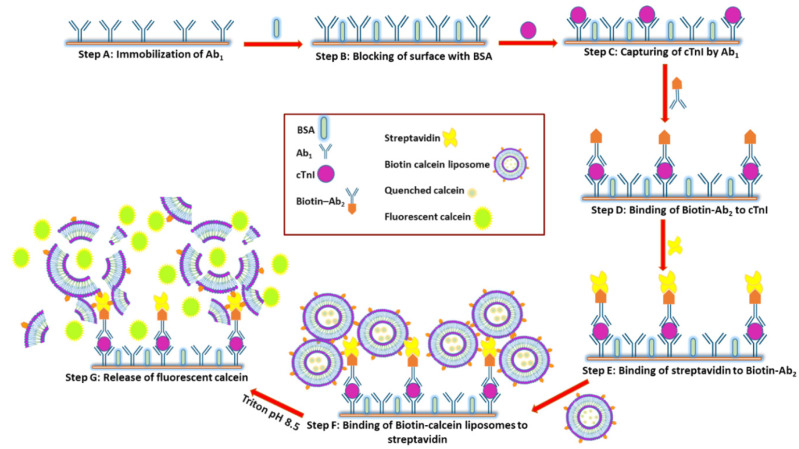
Schematic illustration of cTnI detection via liposome-based fluorescent immunoassay.

Therefore, this paper reports the development of a novel liposome-based signal amplification fluorescent immunoassay for the detection and quantification of cTnI. The approach of this study is based on a fluorescent sandwiched immunoassay (Scheme 1, Step A to Step G) for capturing cTnI between a surface-immobilized capturing antibody and a biotin-conjugated secondary antibody (detection antibody). The detection of the captured biomarker is then achieved via a biotin-conjugated liposome through a streptavidin (SA) intermediate. The liposome acts as the nanocarrier for a load of a fluorescent dye (calcein), which is released upon the lysing of the liposome with Triton X-100 [43,44]. The intensity of the emission signal is dependent on the concentration of the cTnI in the sample.

## 2. Results and Discussion

### 2.1. Characterization and Stability Analysis on Biotin–Calcein Liposomes

Liposomes are non-toxic, biocompatible nano-vehicles formed by the self-assembly of phospholipids into a bilayer, creating an inside aqueous compartment that can be loaded with water-soluble compounds, such as fluorescent dyes (Scheme 1). The outside layer of the phospholipid can be chemically functionalized to be brandished with different moieties, such as ligands or antibodies [45,46,47,48,49]. On the other hand, calcein is a water-soluble fluorescent dye that has a self-quenching property at high concentrations, and it can be loaded inside liposomes at high concentrations [46,50]. The exposure of liposomes to mechanical stress, such as ultrasound waves, or to lysing detergents (Triton X-100), leads to the disintegration of the lipid bilayer and thereby releases the components from the internal compartment of the liposomes [50]. The release of calcein from liposomes in such conditions ends self-quenching and induces a strong fluorescence signal.

**Figure 1:**
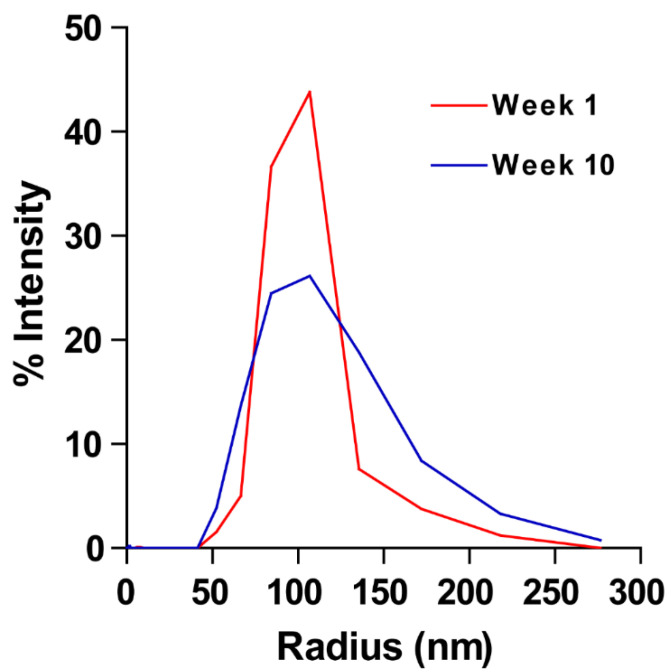
Light-scattering measurements on calcein-loaded liposome. DLS of newly synthesized calcein liposomes and after 10 weeks of storage at 4 °C of the same batch.

The liposomes are a crucial component of the developed assay; hence, the preparation and characterization procedures of these nanocarriers are essential to ensure efficacy and reproducibility. Biotin-conjugated calcein-loaded liposomes were synthesized via the thin-film hydration method, similar to the previously reported studies [45,51], and a full characterization process was applied, including a Stewart assay, to quantify the total lipid concentration of the liposome solution, DSL measurements, and stability studies. The Stewart assay was conducted according to previously reported procedures [51], indicating a calculated total lipid concentration of 10.1 mmol/L. In addition, dynamic light scattering (DLS) studies revealed the size range and % polydispersity (Pd) of the liposomes (Figure 1). The obtained Pd index value was found to be acceptable as it falls in the criteria of its upper limit of 20% for DLS measurements [51]. As shown in Table 1, even after 10 weeks of storage at 4 °C, the liposome solution was found to be stable, with minimal variation in their average radius (96.3 ± 3.9 nm), while maintaining a quenched load of calcein that was released upon the addition of Triton X-100, leading to a five-fold increase in the fluorescence signal.

### 2.2. Standardization of Immunoassay Parameters

A series of experiments were carried out to standardize the conditions of the assay and to optimize the parameters, including the combination of capture–detection antibodies, concentration of Ab_1_, buffer systems (Ab-coating), microplate blocking solution, number of washings in assay steps, assay incubation time, dilutions and concentrations of each assay component, and fluorescence measurement parameters.

#### 2.2.1. Optimization of Capture Antibody and Detection Antibody Combination

The binding of the protein by the detection and the capture antibody needs to take place at spatially distant epitopes to minimize the possible steric effect on the binding efficacy. The cTnI protein has 209 amino acid residues with a unique sequence at N-terminal end (~40 residues) for cTnI over other forms of troponin [52,53]. We have tested three pairs of antibodies specific to different epitopes in order to determine the most efficient combination that provides a strong signal: i) Anti-h cTnI 9701 binds to the epitope at residues 85–95, Anti-h cTnI 9703 recognizes the epitope at residues 39–50, and Anti-h cTnI 9705 binds at 21–30 residues.

**Figure 2:**
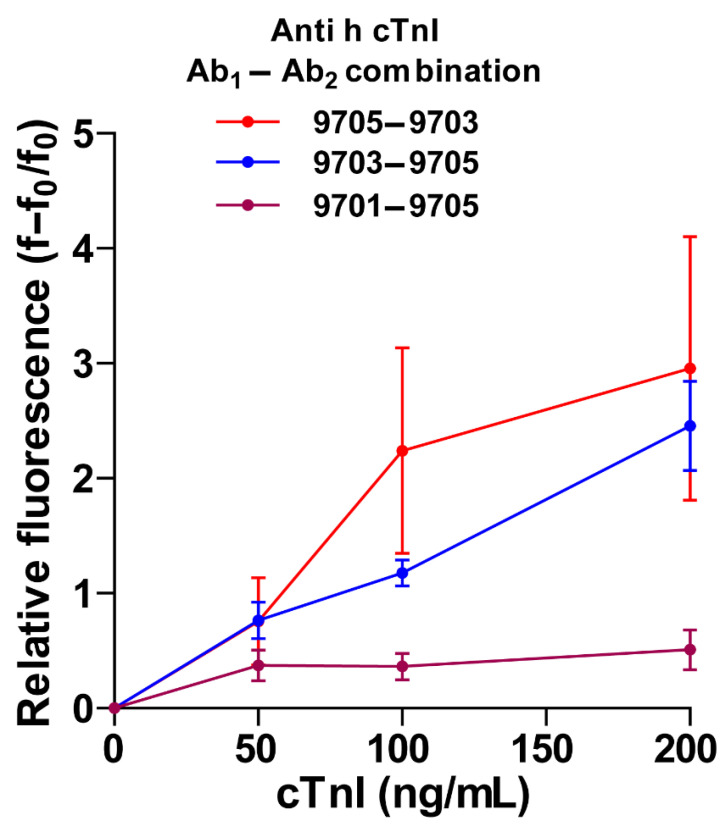
Effect of capture–detection antibody on the assay. The different plots correspond to the change in relative fluorescence over different cTnI levels exhibited by the assays with three different combinations of Ab_1_–Ab_2_. The assays were performed in 3 batches maintaining the coating concentration as 2 µg/mL with cTnI levels of 0, 50, 100, and 200 ng/mL. The experiment used 2 µg/mL of Streptavidin, 2 µg/mL of Biotin–Ab_2_, and 1:100 diluted biotin calcein liposomes. The error bars show the standard deviation of values for the triplicate readings for three different assay experiments. (Two-way ANOVA test showed that there is statistically significant difference (*p* < 0.05) between 9701–9705 assay and the other two assays (*p* = 0.009 and *p* < 0.001), but there is statistically insignificant difference (*p* = 0.446) between 9703–9705 and 9705–9703).

Figure 2 shows a comparison of the relative fluorescence obtained to cTnI levels for the assay trials with the different combinations of Ab_1_–Ab_2_ (9705–9703, 9703–9705, and 9701–9705). The relative fluorescence was calculated for each data point using the relation (f − f_0_)/f_0_, where ‘f’ is the fluorescence emission intensity measured from a well containing a particular cTnI level and ‘f_0_′ is the same for a well with 0–cTnI. Among the three Ab_1_–Ab_2_ combinations tested, the 9705–9703 antibody combination (Figure 2, red plot) presented a good relative increase in the fluorescence for the cTnI levels tested. Based on the results, the combination of Anti-h cTnI 9705–9703 was selected as the capture–detection Ab system for further assay runs. The choice of 9705 ensures a high specificity for capturing cTnI over other forms of troponin since its epitope lies within the unique N-terminal end of the protein.

#### 2.2.2. Effect of Incubation Time

**Figure 3:**
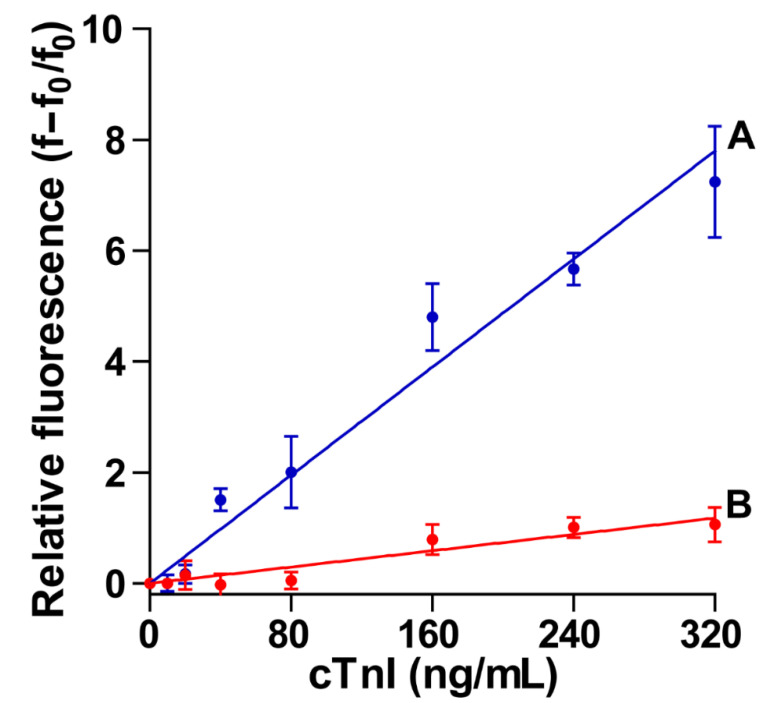
Standardization of assay incubation time. Assay trials were done by varying the incubation time for each step addition; (A) cTnI and Ab_2_–biotin for 60 min and other components (SA and calcein liposome) for 30 min; (B) cTnI and Ab_2_–biotin were incubated for 30 min and other parameters (SA, blocking liposome, and calcein liposome) for 15 min. Less response was observed for B compared to A. Assay conditions used were as follows: coating antibody concentration of 5 µg/mL, cTnI levels of 0–320 ng/mL, 2 µg/mL of SA, 2 µg/mL of Biotin–Ab_2_, and 1:100 diluted biotin calcein liposomes. The error bars show the standard deviation of values for the triplicate readings obtained for three different assay experiments. (Two-way ANOVA test showed that there is statistically significant difference (*p* < 0.05) between A and B assays (*p* < 0.001) and that the change in the relative fluorescence for each assay is significantly dependent (for A, *p* < 0.001; for B, *p* < 0.001) on the concentration of cTnI in solution).

One of the major hurdles in applying sandwich assays in point-of-care testing is the time required to complete the assay. Thus, we investigated whether the decrease in the incubation time of each assay step to half would affect the efficacy of the assay. We performed assay trials with 9705 as a capture Ab_1_ (5 µg/mL) using a shorter incubation time of each assay step such that cTnI and Ab_2_–biotin were incubated for 30 min while other parameters (Streptavidin (SA), blocking liposome, and calcein liposome) were incubated for 15 min. As depicted in Figure 3, the change in the relative fluorescence of the assay with reduced time presented a very low response and linearity correlation (y = 0.0028x, R^2^ = 0.56) to the concentration of cTnI in solution as compared to the assay with double the incubation time of each step, which exhibited a good linear fit (y = 0.0227x, R^2^ = 0.95).

#### 2.2.3. Standardization of Coating Antibody Concentration

**Figure 4:**
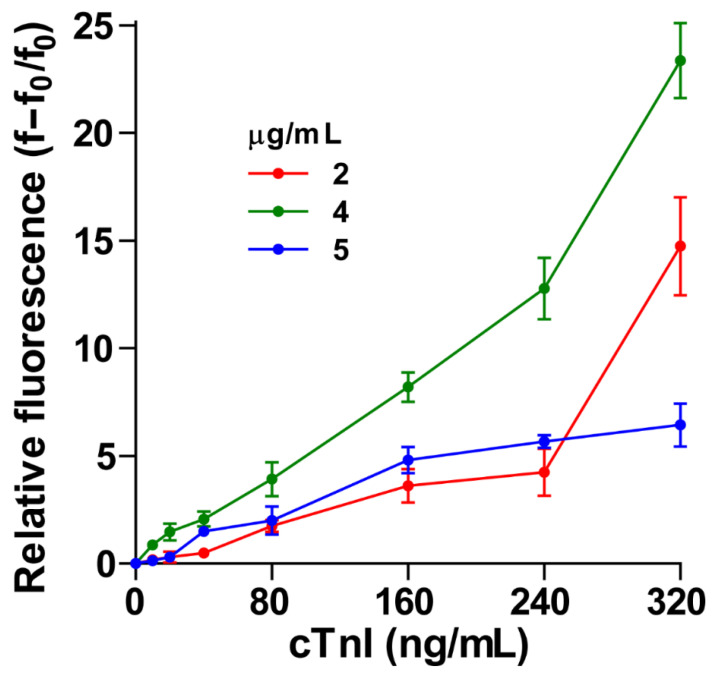
Effect of Ab_1_ (capture antibody) concentration on cTnI assay. The relative emission at different levels of cTnI is plotted for three different concentrations of capturing antibodies (Anti 9705- 2, 4, and 5 µg/mL) keeping other assay parameters the same (cTnI levels 0–320 ng/mL, 2 µg/mL of SA, 2 µg/mL of Biotin–Ab_2_, and 1:100 diluted biotin calcein liposomes). The error bars show the standard deviation of values for the triplicate readings obtained for three different assay experiments. (Two-way ANOVA test showed that there is statistically significant difference (*p* < 0.05) between **4** assay and the other two assays (*p* < 0.001 and *p* < 0.001), but there is statistically insignificant difference (*p* = 0.069) between **2** and **5** assays).

The coating concertation of the capture antibody on the surface can have a detrimental effect on the assay results. A high concentration can lead to the overcrowding of the antibody on the surface and could result in a lower capturing efficacy, while a low concentration could lead to lower sensitivity and a weak signal of the assay. Thus, in order to optimize the coating concentration of the capturing antibody, we selected three concentrations at 2, 4, and 5 µg/mL. The assays were then conducted with all the other conditions maintained, and the relative change in fluorescence (f − f_0_/f_0_) was monitored as the concentration cTnI (ng/mL) was increased. It was noted (as depicted in Figure 4) that an Ab_1_ concentration of 4 µg/mL (green plot) yields the maximum relative fluorescence to the levels of cTnI among the three coating concentrations.

#### 2.2.4. Effect of Streptavidin Concentration and Liposome Dilution

**Figure 5:**
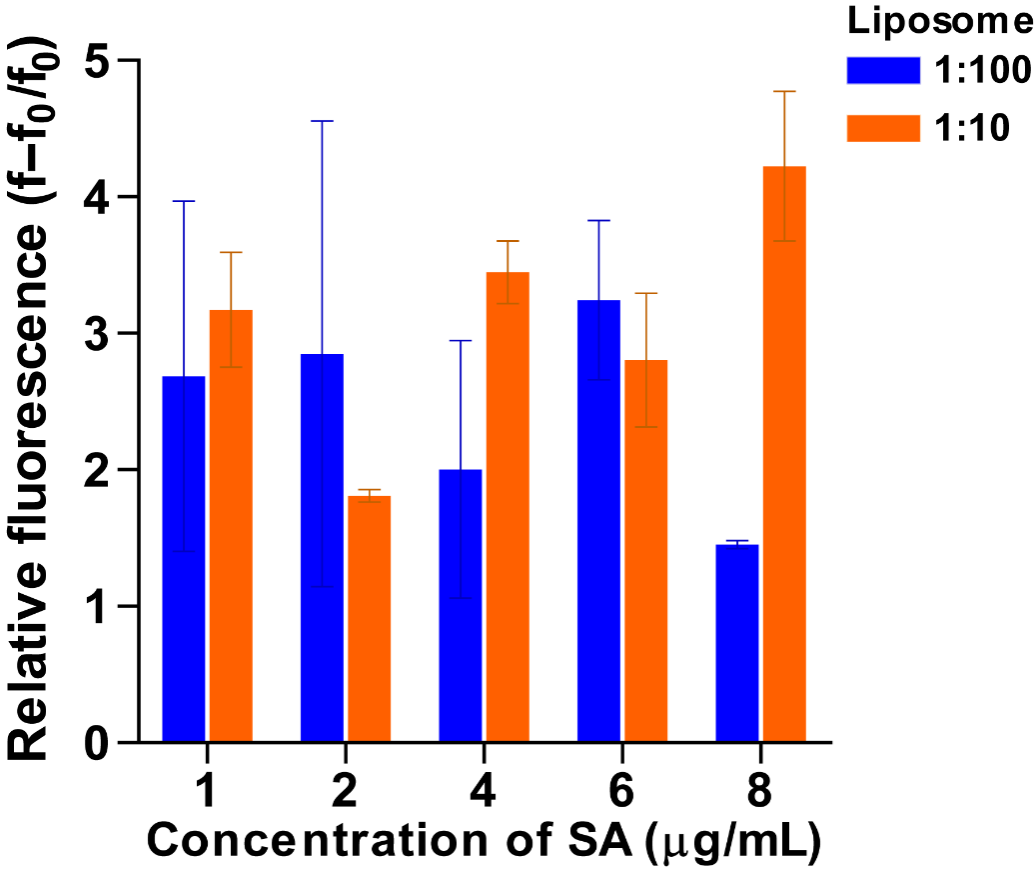
Effect of SA and liposome levels on cTnI assay. The relative emission at different concentrations of SA (1, 2, 4, 6, and 8 µg/mL) is plotted at two different dilutions of calcein liposome (1:100 and 1:10). The assay was performed at a fixed cTnI level of 80 ng/mL, and 2 µg/mL of Biotin–Ab_2_. The error bars show the standard deviation of values for triplicate observations. (Two-way ANOVA test showed that there is statistically insignificant difference (*p* = 0.065) between the two assays and that the change in the relative fluorescence is insignificantly dependent (*p* = 0.710) on the concentration of SA in solution).

The concentrations of the SA and liposomes have a critical and a direct effect on the fluorescence signal of the assay. Too high of a concentration of SA or liposomes can lead to non-specific binding and, hence, a higher fluorescent signal that is not correlated with the concentration of the cTnI in solution. Thus, we conducted a single batch experiment for a fixed level of cTnI (80 ng/mL) at varying concentrations of SA (1, 2, 4, 6, and 8 µg/mL) and calcein-loaded liposome (1:10 and 1:100 dilutions). The observed trend showed no linear correlation of (f − f_0_/f_0_) with the SA concentration or liposome concentration/dilution. Thus, concentrations of SA at 2 µg/mL and liposome dilution at 1:100 were used as the optimized concentration for the standardized assay (Figure 5).

#### 2.2.5. Standardized Assay for the Detection of cTnI Spiked in Bovine Serum Albumin (BSA)/Phosphate Buffer Saline (PBS)

As the variant conditions of the assay were optimized, we conducted the standardized assay in a physiological buffer system (BSA/PBS at pH 7.4) to validate the feasibility of the developed assay before testing the sensitivity towards the cTnI in the human serum. The cTnI was spiked into the buffer solution at various concentrations, and the assay was conducted at the following optimized conditions:

(i) Concentrations: coating Ab_1_ concentration––at 4 µg/mL, cTnI levels: 0–320 ng/mL, SA:2 µg/mL, Biotin–Ab_2_:2 µg/mL, and 1:100 diluted biotin calcein liposomes
(ii) Incubation time: cTnI and Ab_2_–biotin for 60 min, SA and calcein liposome for 30 min, and blocking liposome for 15 min.

**Figure 6:**
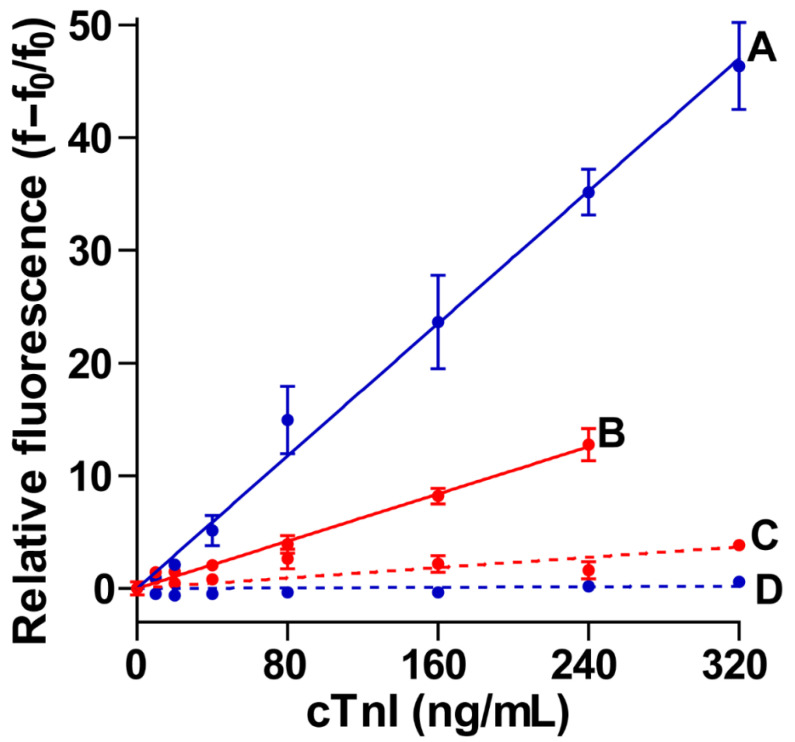
Comparison of the standardized assay (cTnI in BSA/human serum) with their reference assays. The relative emission at different levels of cTnI is plotted to a wide range of cTnI levels for (A) standardized immunoassay with cTnI-human serum and (B) standardized immunoassay with cTnI–BSA/PBS. C and D (dotted pattern plots) correspond to the reference assay for B and A, respectively. The reference assay indicates the standardized assay done in the absence of Ab_1_ (microplate wells coated with 2% BSA instead of capturing antibodies were used as assay platform). Error bars represent the standard deviation of two independent triplicates. (Two-way ANOVA test showed that there is statistically insignificant difference (*p* < 0.05) between the two independent assays for each of serum and PBS (for A, *p* = 0.533; for B, *p* = 0.795), and the change in the relative fluorescence for each assay is significantly dependent (for A, *p* < 0.001; for B, *p* < 0.001) on the concentration of cTnI in solution).

As the reproducibility of an immunoassay is an important criterion for practical applications, we conducted two independent batches of assays with triplicate runs. The assay was found to sense a wide range of cTnI, exhibiting a good linear fit for the cTnI range 0–240 ng/mL (y = 0.0525x) with a correlation coefficient of R^2^ = 0.98 (Figure 6, plot B). The assay response was found to have a high signal-to-noise ratio as compared to its reference assay values (Figure 6, plot C) with a limit of detection (LOD) at 22.5 ng/mL determined as the minimum concentration of cTnI at which the mean (f − f_0_) values over all the replicates is larger than (f_0_) by at least three standard deviations of the negative controls.

#### 2.2.6. Quantification of cTnI in Human Serum

The next step was to assess the efficacy of the liposome-based fluorescent immunoassay in the quantification of the cTnI in the human serum. This is particularly important to determine the potential of applying the assay in practical clinical settings. Thus, we ran the assay on cTnI samples dissolved in undiluted human serum over a concentration range of cTnI 0–320 ng/mL, as per the optimized conditions used for cTnI in the BSA/PBS buffer system. The change in the relative fluorescence signal versus the change in the concentration of cTnI is depicted in Figure 6, plot A. The results show a linear relationship (y = 0.1468x) of the relative fluorescence to the cTnI levels in the serum with a very high correlation (R^2^ = 0.994). It was interesting to note that the cTnI-serum assay ended up in a higher response (Figure 6, plot A) and even higher signal-to-noise ratio versus the reference as compared to the assay conduced in the BSA/PBS system (Figure 6, plot B). This can be attributed to the higher stability and solubility of cTnI in blood serum than in BSA/PBS [54]. Moreover, the assay has shown roughly around a 45-fold increased response for the cTnI-serum samples (320 ng/mL) versus those of the reference system (Figure 6, plot D), which has all the components of the assay except that the wells were coated with BSA instead of Ab_1_. The limit of detection (LOD) of the assay was 6.5 ng/mL (determined as the minimum concentration of cTnI at which the mean (f − f_0_) values over all replicates is larger than (f_0_) by at least three standard deviations of the negative controls), which is very close the normal level (5 ng/mL) cut-off in a patient’s blood. Although our results presented a higher value of LOD than the previously reported assays [2,24,55,56,57], the assay was highly specific towards cTnI even in the presence of other interference proteins in the human serum and, more importantly, it has a wide linear dynamic range from right above the normal concentrations (LOD) of cTnI to 320 ng/mL.

## 3. Materials and Methods

### 3.1. Chemicals/Materials

1,2-Dipalmitoyl-sn-glycero-3-phosphocholine (DPPC), 1,2-Diastearoyl-sn-glycero-3-phosphoethanolamine-n-[biotinyl(polyethylene glycol)-2000] (DSPE–PEG (2000) Biotin), Cholesterol and 1,2-diastearoyl-sn-glycero-3-phosphoethanolamine-n-[methoxy(polyethylene glycol)-2000] (DSPE–PEG (2000) and 200-nm polycarbonate filters were purchased from Avanti Polar Lipids, Inc., (Alabaster, AL, USA). Recombinant cTnI antigen and cTnI Antibodies [Anti-h cTnI 9705 SPTN-1, Anti-h cTnI 9703 SPTN-5 and Anti-h cTnI 9701 SPTN-5] from Medix Biochemica (Espoo, Finland). NHS-dPEG 4-biotin (Quanta Biodesign), Streptavidin from *Streptomyces avidinii*, H4522-Human serum type AB (Male) Maxisorp-NUNC immune plate (Thermo scientific, Waltham, MA, USA), PD-10 Sephadex TMG-25 M (GE Healthcare, Chicago, IL, USA), Calcein, SephadexTM-G-100 (GE-Healthcare), Dialysis tubing (CAROLINA Dialsis tubing, Molecular cut off 13,000, Burlington, NC, USA), Triton ^TM^ X-100, n,n-Dimethylformamide, PBS Tween ^TM^-20, phosphate buffered saline tablet, borate buffer (pH 8.5), sodium bicarbonate buffer (pH 9.4), and all other chemicals used for the study were of high analytical grade and were purchased from Sigma-Aldrich/Merck (Baden-Württemberg, Germany).

### 3.2. Major Instrumentations

The synthesis of liposomes was done with the help of Stuart Rotary evaporator. Sonication bath (Elma D-78224, Melrose Park, IL, USA) was used to get unilamellar vesicles (liposomes). Avanti Polar Lipids, Inc extruder was used for liposome-extrusion. The average radius and % polydispersity of liposomes were examined by dynamic light scattering analysis (DLS) using Dyanopro Nano star Laser Photometer (Wyatt Technology Corp., Santa Barbara, CA, USA). Fluorescence emission measurements were done with the help of FLSP920 series of fluorescence spectrometers (Edinburgh Instruments Ltd., Livingston, UK). UV-Visible Spectrophotometer (EVOLUTION 60 S, Thermo Scientific) was used in Stewart assay for measuring the absorbance. FLUOstar Omega Plate reader from BMG LABTECH was employed for 96-well microtiter plate fluorescence measurements.

### 3.3. Preparation of Dye (Calcein) Solution

The study used calcein as a fluorescent dye considering its excellent fluorescent properties reported [50,58]. Calcein solution (100 mM) was prepared in deionized water, and the pH of the solution was adjusted to 7.4 with 1 N NaOH.

### 3.4. Synthesis of Calcein-Loaded Biotin–Liposome

Nano liposomes were synthesized via the thin-film hydration method [45,46]. Briefly, the lipids formulation of 70 mol% DPPC, 2.3 mol% DSPE–Biotin, and 27.7% cholesterol were dissolved in chloroform (4 mL) by proper vortexing and sonication in a 100 mL round bottom flask. The lipid solution was carefully evaporated using a rotary evaporator at a low vacuum for the first 10 min with a rotation speed of 120 rpm at 25 °C. The film was dried for another 50–60 min under a high vacuum. Liposomes were synthesized by hydration of the dry lipid film at 65 °C with 1.5 mL of calcein solution. Adequate vortexing and sonication were done during and after the hydration process. The large multilamellar vesicles of various sizes then passed through an extruder (Avanti Polar Lipids, Alabaster, AL, USA) 12–15 times through 0.2–μm polycarbonate membranes at 65 °C to yield a homogeneous solution of unilamellar vesicles. The extruded biotin-conjugated liposomes were then purified by a double column elution chromatography using Sephadex G-100 resin (10 mL), which was pre-equilibrated in PBS (7.4). The liposome was then stored at 4 °C for further use.

### 3.5. Synthesis of Blocking Liposome

Lipid film formation and hydration steps followed the same steps used for calcein–biotin liposome synthesis. The ratio of lipids and chloroform used was the same but DSPE–Biotin was replaced with DSPE–PEG and hydration of lipid thin film was done with 1.5 mL of PBS (pH 7.4). Adequate vortexing and sonication were done during and after the hydration process for nearly 10 min. Blocking liposomes were purified by a spin column elution (PD-10 column), which was equilibrated with PBS (pH 7.4) at 3000 rpm for 3 min.

### 3.6. Characterization and Stability Analysis on Liposomes

#### 3.6.1. Dynamic Light Scattering (DLS) Measurements

The size range and % polydispersity of Biotin–calcein liposomes were measured by DLS analysis. The liposome solutions were diluted around 100 times before DLS analysis. Regular DLS was done to monitor the size variation to confirm the stability of liposomes under the storage temperature of 4 °C.

#### 3.6.2. Fluorescence Spectroscopy

The fluorescence emission profiles of diluted calcein liposomes (1:10^5^ in PBS) were measured using a fluorescence spectrometer. The emission spectra were taken in the absence and presence of 5% Triton X-100 in Borate buffer (pH 8.4) at a scanning excitation wavelength of 490 nm and an emission wavelength range of 500–700 nm.

#### 3.6.3. Quantification of Lipid Content in Biotin Calcein Liposome

The phospholipid concentration of the liposome solution was colorimetrically determined by Stewart assay as described in the literature [51]. Around 50 µL of undiluted liposome fraction in a round bottom flask was placed in the rotary evaporator and dried up the suspended medium under vacuum. After drying, the flask was added with 1 mL of chloroform and was sonicated until a clear solution was formed. Varying volumes of solution from the flask were transferred to centrifuge tubes and made the total volume of solution to 2 mL with chloroform followed by the addition of 2 mL of ammonium ferrothiocyanate. The solutions were mixed properly and centrifuged for 10 min at 1000 rpm. The top dark layer was carefully discarded without touching the bottom layer and the clear chloroform bottom layer was moved to a quartz cuvette. The optical density of the cuvette solution was measured using an ultraviolet-visible spectrophotometer at λ_max_ = 485 nm against chloroform-assay solution as blank.

### 3.7. Conjugation of Biotin-Secondary Antibody

The tethering of NHS-dPEG 4-biotin (around 57 mM in DMF) to secondary/detection antibody (Ab_2_) [pre dialyzed in PBS (pH 7.4)] was accomplished by reacting them in 20:1 volume ratio for 2 h at room temperature in a glass vial, a modified protocol reported earlier [55]. After conjugation, the excess unconjugated biotin was removed by spin column (equilibrated with PBS (pH 7.4) containing 0.01% NaN_3_) elution done at 3000 rpm for 3 min. The Biotin–Ab_2_ solution was stored at 4 °C for further use.

### 3.8. Standardization of Immunoassay Parameters

#### 3.8.1. Choice of Assay Platform

For the fluorescent assay, the Ab_1_ was covalently immobilized on the surface of activated microplates (Maxisorp NUNC 96 microplates, Thermo Fisher Scientific, Kamstrupvej, Denmark) through a covalent bond linkage of the activated carboxylate surface and an amine group of the antibody. The microplate wells provided the container for the successive addition and washing of assay reagents and an easy way for sensing and quantification of fluorescence intensity via microplate reader.

Preliminary standardization steps were performed to confirm the reproducibility and consistency of results while minimizing the time and cost to run the assay. Assay trials were carried out for selecting the best combinations of Capturing (Ab_1_)-Detection (Ab_2_) cTnI-antibodies, optimizing the assay conditions, such as incubation time, the number of washings, dissolving buffer systems, capturing antibody concentrations, the concentration of Streptavidin, dilutions of liposomes, etc.

#### 3.8.2. Optimization of Capturing (Ab_1_)-Detection (Ab_2_) cTnI-Antibodies Combination

As Anti-h cTnI 9705 SPTN-1 and Anti-h cTnI 9703 SPTN-5 were selected as detection antibodies (conjugated with Biotin), their best complimentary antibodies, Anti-h cTnI 9701 SPTN-5, Anti-h cTnI 9703 SPTN-5, and Anti-h cTnI 9705 SPTN-5, were opted as capturing antibodies (for plate immobilization). Assay trials were carried out with different combinations of Ab_1_–Ab_2_ (Anti-h cTnI 9705–9703, 9703–9705, and 9701–9705). The Ab_1_—antibodies were dissolved in bicarbonate buffer (pH 9.4) to get a final concentration of 2 µg/mL. The assay was performed for all the mentioned combinations as per the protocol mentioned in Section 3.9, with cTnI levels of 0, 50, 100, and 200 ng/mL.

#### 3.8.3. Effect of Incubation Time

The combination of Anti-h cTnI 9705 SPTN-1 and Anti-h cTnI 9703 SPTN-5 was found to be more efficient and was used for further assay optimization steps. Assay with wells coated at 5 µg/mL concentration of Ab_1_ (9705) (Section 3.9) was performed with reduced (half) incubation time to confirm whether the lower incubation assay reaction gives better results. cTnI and Ab_2_—biotin incubated for 30 min and other parameters (SA, Blocking liposome, and calcein liposome) for 15 min.

#### 3.8.4. Effect of Capturing Antibody (Ab_1_) Concentration

To fix the best coating concentration of Ab_1_, the assay was tried with three different Ab_1_ concentrations 2, 4, and 5 µg/mL and followed the normal assay steps as per Section 3.9.

#### 3.8.5. Effect of SA Concentration and Liposome Dilution

To confirm the selected SA and calcein liposome concentration as best, two sets of assays were performed for a fixed cTnI level of 80 ng/mL. The assay used 1:10 and 1:100 dilutions of liposomes (total lipid concentration = 10.1 mM) for SA levels of 1, 2, 4, 6, and 8 µg/mL, maintaining the other conditions same as reported in Section 3.9.

### 3.9. Standardized Assay Protocol (cTnI in Physiological Buffer System)

For the standardized assay, 100 µL of Ab_1_ (Anti-h cTnI 9705 SPTN-1 dissolved in bicarbonate buffer (pH 9.4)) at 4 µg/mL were loaded into test wells, whereas 2% BSA/PBS (300 µL) were coated in control wells. The plate was incubated at 4 °C overnight for maximum binding. After washing, the plate was blocked with 2% BSA (300 µL) for 2 h at room temperature, shaking at 100 rpm. The solution was removed and wells were washed twice with PBST and one time with PBS and then tapped on tissues. Both control and Ab_1_-coated wells were coated with 100 µL of varying concentrations of cTnI antigen (0, 10, 20, 40, 80, 160, 240, and 360 ng/mL, diluted in BSA/PBS (1%)) and incubated at 37 °C for 1 h, shaking at 100 rpm. The wells were washed 3 times with PBST (300 µL) and tapped on tissues. 100 µL of Biotin–Ab_2_ (2 µg/mL, diluted in BSA/PBS (1%) was loaded to all wells and incubated at 37 °C for 1 h, shaking at 100 rpm followed by washing the plate 3 times with PBST (300 µL). The wells were then incubated with 100 µL of Streptavidin (2 µg/mL, diluted in BSA/PBS (1%) at room temperature for 30 min, shaking at 100 rpm, and washed 3 times with PBS (300 µL). The wells were then blocked with 100 µL of 1:100 times diluted blocking liposome (PBS) and allowed to incubate for 15 min at room temperature at same shaking conditions. After incubation, the solution was removed carefully and again incubated with calcein liposomes (100 µL of 1:100 times diluted in PBS) for 30 min at room temperature/shaking. Liposomes were removed and the plate was washed 5 times with PBS (300 µL) and tapped on tissues after each wash. The fluorescence of dry wells was then measured with the help of FLUOstar Omega at an excitation wavelength of 490 nm and emission wavelength of 520 nm with a machine Gain of 1000. All the wells were then added with 100 µL of 5% Triton X-100 (Borate buffer, pH 8.5) and allowed to mix for 10 min for the proper lysis of liposomes. The fluorescence emission from each well was measured at the same conditions as mentioned before. The relative increase in fluorescence intensity was found to be directly proportional to cTnI levels in the wells. The assays were conducted in week 4, 5, and 10 after preparation of the liposomes.

A control assay (reference) was conducted to analyze the sensitivity and signal noise (non-specific binding) effects on the assay. The assay was conducted in microtiter wells coated 2% BSA (instead of Ab_1_) with all other steps being the same and the standardized steps described above.

### 3.10. cTnI Detection in Human Serum

The performance of immunoassay was tested in human serum type AB (male), spiked with different levels of cTnI. The efficacy and sensitivity of the assay were analyzed by comparing the results with those of the reference plate (assay conducted in 2% BSA coated plate). Same standardized assay steps were followed for the detection of cTnI in human serum. The assay was conducted in week 10 after preparation of the liposomes.

### 3.11. Statistical Analysis

Statistical significance was assessed using Microsoft Excel software. A *t*-test or a two-way ANOVA test were used to analyze the experimental data with statistically significant difference set at *p* < 0.05. All error bars are a standard deviation from the mean values of three experiments.

## 4. Conclusions

In summary, we established a rapid and sensitive detection system for cardiac troponin I in the physiological buffer system and biological fluid (serum) by a liposome-based fluorescent amplification method. The calcein-loaded liposomes have featured reasonable stability and fluorescent properties over several weeks, which met the criteria of an efficient fluorophore nanocarrier for the assay. The proposed assay exhibited a noble analytical performance with dynamic linearity for human serum spiked with cTnI at a wide range of concentrations. In addition, it presented high selectivity to cTnI among other interfering proteins, such as BSA and other serum proteins. The wide range of concentration indicates that this immunoassay may have a promising potential for the direct application on non-diluted patients’ serum samples since the LDR falls within the relevant clinical concentrations of cTnI. This feature could potentially reduce the time of diagnosis and may provide information on the timing of MI incidents based on the level of cTnI in the blood using a single experiment without the need for repeated testing. Yet, further optimization studies with regard to assay time and a change in the immobilization platform would be required to develop the assay to the level of a point-of-care kit. Besides, developing such a kit for clinical settings requires further validation and testing with clinical samples of patients’ serum and benchmarking against other developed kits in the market.

## Figures and Tables

**Table 1:** Dynamic light scattering measurements of calcein liposomes.

Calcein Liposome	Mean Radius (nm)	%Pd
Freshly Synthesized	98.2 ± 2.4	12 ± 3.8
After 10 weeks	96.3 ± 3.9	15.9 ± 1.5

Values are the mean of three independent measurements. (Statistical analysis using *t*-test showed insignificant differences between the mean radius values of week 1 and week 10 (*p* > 0.05)).

## Data Availability

Not applicable.

## Sample Availability

Samples of the compounds are not available from the authors.
